# Raman-based detection of hydroxyethyl starch in kidney allograft biopsies as a potential marker of allograft quality in kidney transplant recipients

**DOI:** 10.1038/srep33045

**Published:** 2016-09-09

**Authors:** Vincent Vuiblet, Michael Fere, Ezechiel Bankole, Alain Wynckel, Cyril Gobinet, Philippe Birembaut, Olivier Piot, Philippe Rieu

**Affiliations:** 1 UMR CNRS 7369 MEDyC, Université de Reims Champagne-Ardennes, Reims, France; 2 Nephrology division, Maison Blanche University Hospital, Reims, France; 3 Biopathology Laboratory, Maison Blanche University Hospital, Reims, France; 4 Department of Anesthesia, Maison Blanche University Hospital, Reims, France; 5 PICT (Cellular and Tissular Imaging Platform), Université de Reims Champagne- Ardenne, Reims, France

## Abstract

In brain-dead donor resuscitation, hydroxyethyl starch (HES) use has been associated with presence of osmotic-nephrosis-like lesions in kidney transplant recipients. Our aim was to determine whether the presence of HES in protocol renal graft biopsies at three months (M3) after transplantation is associated with renal graft quality. According to the HES administered to the donor during the procurement procedure, two groups of patients were defined according graft exposition to HES: HES group, (N = 20) and control group (N = 6). Detection and relative quantification of HES was performed by Raman spectroscopy microimaging on M3 protocol renal graft biopsies. Statistical analyses were used to investigate the association between Raman data and graft characteristics. HES spectral signal was revealed negative in the control group, whereas it was positive in 40% of biopsies from the HES group. In the HES group, a stronger HES signal was associated with a lower risk of graft failure measured by the Kidney Donor Risk Index (KDRI) and was correlated with the allograft kidney function. Thus, HES accumulation in donor kidney, as probed by Raman biophotonic technique, is correlated with the quality of donor kidney and consequently the graft renal function and graft survival.

The shortage of organs is a major limitation for transplantation, making the care of potential organ donors an important issue. Optimizing medical management of brain-dead donors may increase the number of eligible donors and improve organ function for the purposes of transplantation^1^,^2^. Adequate fluid resuscitation is a major step in the predefined donor management goals, because circulatory instability often occurs after neurologic death^3^. Restoration of intravascular volume requires filling with crystalloid or colloid solutions. Among the colloid solutions used is hydroxyethyl starch (HES), which is produced by hydrolysis and hydroxyethylation of amylopectin, a highly branched starch that is obtained from waxy maize or potatoes. Early forms of this solution had a high molecular weight (200 kd) and a high degree of substitution (0.5 or 0.6), and were associated with both renal dysfunction and increased risk of bleeding^4^,^5^. More recently, “third-generation” HES, so-called tetrastarch (molar degree of substitution 0.4 and medium molecular weight of 130 kDa), has also been associated with increased mortality, renal dysfunction and an increased risk of bleeding in critically ill adult patients, in patients with pre-existing renal dysfunction and in patients undergoing open heart surgery^6^,^7^,^8^. In brain-dead donor resuscitation, HES use has been associated with osmotic-nephrosis-like lesions in kidney transplant recipients^9^,^10^.

Animal and human studies have shown that HES can accumulate in tissues, and this phenomenon is believed to be potentially harmful^11^. In the kidney, one hypothesis is that HES uptake and accumulation in the proximal tubules contributes to lysosomal alteration of tubular cells leading to osmotic nephrosis lesions^12^.

Raman microscopy is a powerful means of determining the chemical properties of materials with a spatial resolution approaching a micrometer. It is a photonic technique based on the inelastic scattering of light generated by the interaction of monochromatic radiation with a sample. The spectral analysis of the scattered light gives access to the vibrational modes of the molecular constituents of the sample. The coupling of a Raman spectrometer with an optical microscope makes it possible to collect spectra from volumes of the order of 1 μm^3^, enabling the analysis of microscopic features of biological samples. In pharmaceutical research, Raman spectroscopy makes it possible to characterize drugs in biological models such as neoplastic cells or tissues such as skin^13^,^14^,^15^.

We have recently shown that Raman spectroscopy is a candidate biophotonic technique to detect the presence of HES in kidney biopsies originating from patients who received HES during collapse, and who develop acute kidney injury associated with osmotic nephrosis lesions^16^. In the present study, we used Raman spectroscopy to detect, in a non-destructive and label-free manner, the presence of HES in protocol renal graft biopsies performed three months after transplantation from brain-dead kidney donors who received HES solutions. Our objective is to investigate the clinical meaning of Raman detection of HES in protocol renal graft biopsies at three months after transplantation, in terms of renal graft quality. We hypothesize that the accumulation of HES in tubular renal cells could be linked with the functional quality of these cells. Indeed, according to our hypothesis, despite the renal toxicity of HES reported by literature^17^,^18^,^19^, the accumulation of this molecule in tubular renal cells could be a marker of better renal graft quality. In this view, medical criteria currently considered in clinics were compared to the Raman-based HES detection data.

## Results

**Figure 1:**
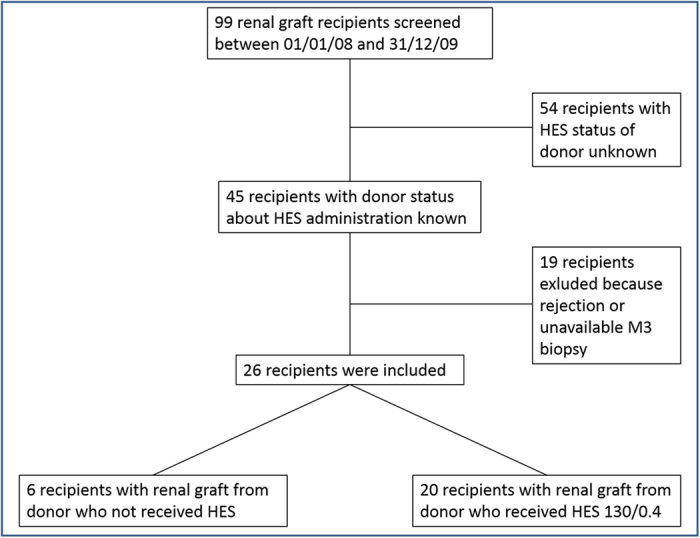
Flow chart of the study design.

In our study, 26 renal transplantations were analysed (Fig. 1): 20 cases for which HES 130/0.4 (VOLUVEN; FRESENIUS-KABI) had been administered in the 72 hours prior to organ retrieval (HES group) and 6 transplantations where no colloid compounds were used (control group). The clinic-demographic characteristics of the brain-dead kidney donors and transplant recipients are shown in Table 1. Biopsies corresponding to these 26 M3 grafts were examined by Raman imaging.

### HES spectral signal in M3 graft biopsies

**Figure 2:**
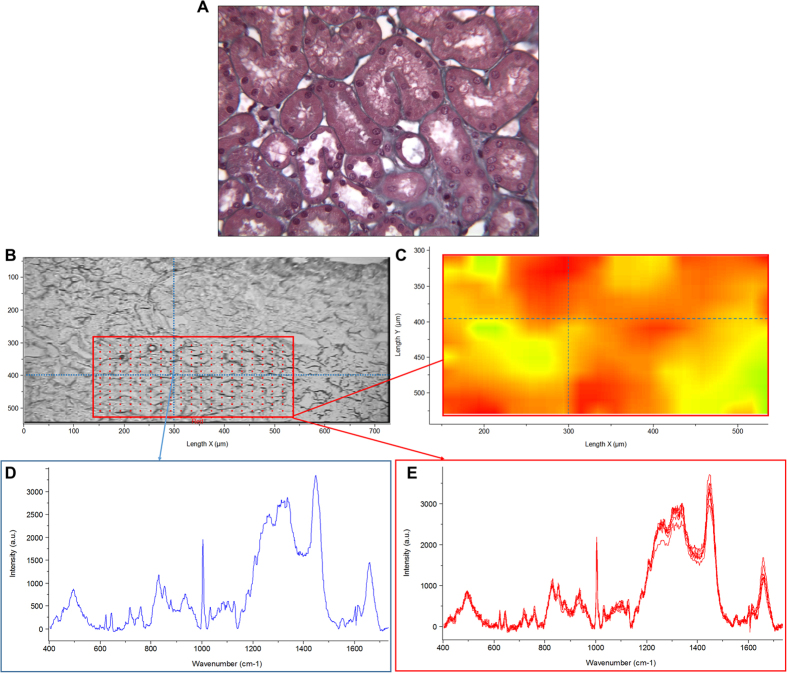
Raman image together with extracted Raman spectra originated from a M3 graft biopsy from the HES group. (**A**) Masson trichrome stained section from paraffin-embedded biopsy depicting a renal tubular sections area (x20) (**B**) 10 μm-thick cryosection of renal graft biopsy observed under white light without staining. Red frame corresponds to the region of interest selected for Raman analysis; red points correspond to spots of Raman measurements (**C**) Raman image constructed from the integrated intensity on the whole fingerprint spectral range. Color scale: yellow to red corresponding to low to high intensity (**D**) Spectrum extracted from one pixel (pointed by the blue arrow) (**E**) Spectra extracted from the selected ROI (red frame).

A Raman image together with extracted Raman spectra originated from a M3 graft biopsy from the HES group is depicted in Fig. 2. Vibrations specific of the protein content such as the amide I band around 1650 cm^−1^ or the ring breathing vibration of the phenylalanine at 1003 cm^−1^ are clearly visible. In addition, HES is highlighted by the vibration at 480 cm^−1^ assigned to a collective mode of the macromolecule skeleton. The fine difference between these spectra was not eye-accessible and require a computer data processing. Indeed, because the Raman spectrum of renal graft parenchyma is a very complex Raman spectrum, the detection and quantification of HES by Raman spectroscopy on these renal graft biopsies were not only based on the analyze of this 480 cm^−1^ band. That is the reason why we used a fitting algorithm to research the HES contribution in tissue considering all the HES spectral and not only one single band. By this mean, we found that the average HES spectral signal intensities for the HES and control groups were 0.21 ± 0.27 and 0.05 ± 0.06 respectively (p = 0.02) (Table 1). For all cases from the control group, the HES spectral signal intensity was lower than the positive threshold (<0.2). Among the HES group, 8 cases presented a HES spectral signal intensity higher than the mean + 2 SD of negative controls (>0.2) meaning that HES compounds can be detected in 40% of renal graft biopsies three months after transplantation from HES-perfused, brain-dead kidney donors. However, histological re-examination of M3 graft biopsies from HES and control groups failed to find tubular vacuolization or osmotic nephrosis-like lesions, suggesting that the presence of HES in kidney tubular sections is not necessarily associated with osmotic nephrosis-like lesions.

### Factors associated with HES spectral signal on M3 graft biopsies

**Figure 3:**
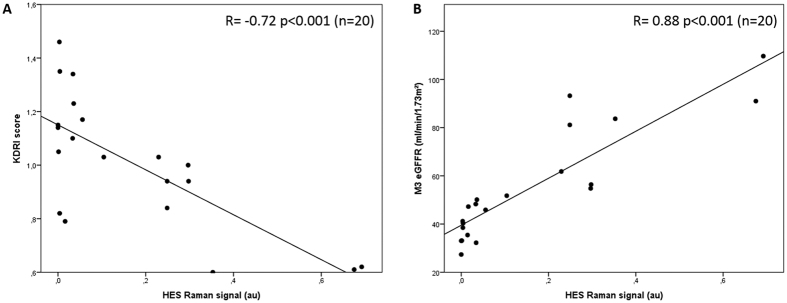
Relationship between HES spectral signal (arbitrary unit; a.u.) and (**A**) the Kidney Donor Risk index (KDRI) (r = −0.72; p < 0.001) and (**B**) estimated glomerular filtration rate at 3 months (eGFR at M3 in ml/min/1.73 m^2^) (r = 0.88, p < 0.001).

Research of correlation between clinical data and Raman-based HES quantification was processed for positioning the biophotonic approach in terms of clinical relevance. Initially, univariate analysis identified recipient age (p = 0.01), donor age (p < 0.001), cause of death (p = 0.03), KDRI (p < 0.001); administered volume of HES (p = 0.002) and eGFR at M3 (p < 0.001) as variables with a significant effect on the detection of HES spectral signal in M3 graft biopsies (Table 2). In addition, the multivariate analysis revealed that only eGFR at M3 (p = 0.006) remained significant. HES infused volume was correlated with HES spectral signal at M3 by univariate analysis but no in multivariate analysis. This fact was probably related to the strong association between HES volume and traumatic cause of death (p = 0.001). Interestingly, based on these statistical data, it is is noteworthy that the HES spectral signal was correlated with KDRI (r = 0.72, p < 0.001) and also logically, since KDRI reflects renal graft quality, a correlation between HES spectral signal and M3 eGFR (r = 0.88, p < 0.001) was highlighted (Fig. 3).

## Discussion

The presence of HES in protocol renal graft biopsies performed three months after transplantation from brain-dead kidney donors who received HES solutions was investigated by Raman spectroscopy. We found that HES compounds could be detected with this label-free technique in 40% of these biopsies, using a methodology previously validated for the detection of tubular accumulation of HES [19]. The absence of a significant HES spectral signal in the control group not exposed to HES argues in favour of the specificity of this technique. In addition, our results show that the accumulation of HES was positively and strongly correlated with the allograft kidney function (r = 0.88 p < 0.001) and inversely correlated with the graft failure risk measured by the Kidney Donor Risk Index (r = −0.72; p < 0.001). These results were obtained from 26 cases. It is noteworthy to mention the difficulty to constitute a cohort for such a study. Indeed, from 99 patients transplanted from brain-dead kidney donors managed during the inclusion period, 54 were excluded due to the lack of complete information regarding HES use. Nineteen more were excluded due to the lack of M3 biopsy (n = 16) or kidney rejection before M3 (n = 3).

Implantation or reperfusion biopsies should have been very interesting to specifically investigate the HES absorption in renal tubular cells. Nonetheless, in this study, we examined M3 biopsies which provide valuable data demonstrating the persistence of HES in tubular cells until 3 months after the transplantation.

In a precedent published work from our team^20^, we described the methodological approach based on Raman spectroscopy, used to detect and quantify HES in kidney. Thus, we report that HES could be detected by Raman micro-imaging in renal biopsies from patients who received this molecule after a haemodynamic failure. The presence of HES in renal tubules detected by Raman micro-imaging with persistent kidney injury is consistent with the renal toxicity of HES actually admitted in literature^17^,^19^. The present study is focused on examination of renal graft, with the novelty to investigate the relationship between the Raman detection of HES and the whole organ function and quality.

Experimental data in animal models have demonstrated that a certain proportion of the HES infused is transiently stored in tissue^11^,^21^,^22^. In humans, tissue deposits of HES have been demonstrated^11^,^23^ especially in the kidney. This HES renal accumulation has been previously revealed by light microscopy showing persistent osmotic nephrosis-like lesions in proximal tubules^9^,^11^. In an isolated porcine renal perfusion model comparing high and low molecular weight HES (200/0.5 and 130/0.4) with Ringer’s lactate, osmotic nephrosis-like lesions of the tubuli were present in all groups, albeit to a lesser degree after administration of Ringer’s lactate solution, demonstrating that these tubular epithelial vacuoles are not necessary HES-containing vacuoles, but can be an independent sign of lysosomal alteration associated with cell damage^24^. Using Raman microspectroscopy, we previously demonstrated for the first time that HES can be detected in renal biopsies from patients who experienced acute renal failure associated with collapse and HES administration, and had biopsy-proven osmotic nephrosis- like lesions^20^. In the present study, we found that the presence of HES in the kidney may also be identified in the absence of osmotic nephrosis-like lesions, showing that the accumulation of HES in the kidney does not necessarily have an impact on the histological aspect of tubular cells as it can be examined by light microscopy.

After HES infusion in humans, the smaller molecules of the HES polydispersal solution whose size is below the renal threshold are excreted first in the urine. Larger molecules are metabolised by plasma α-amylase. An range of 62–68% of the HES (130/0.4) dose perfused is excreted in the urine after 72 hours in healthy volunteers^21^. As for dextran^4^,^25^, filtered HES fragments may be partially reabsorbed from the ultrafiltrate by the cells of the renal proximal tubules by means of pinocytosis, and because the agent is only slowly digestible by lysosomal enzymes, it may remain in the lysosomes of the tubular cells. The amount of HES stored in the kidneys may therefore be dependent not only on the quantity of HES administered, but also on the glomerular filtration rate, and the proximal tubular endocytic traffic rate. Interestingly, we reported that graft HES accumulation was inversely well correlated with the Kidney Donor Risk Index (r = −0.72; p < 0.001). KDRI is a scoring system based on 10 donor factors providing a detailed evaluation of the donor quality of deceased donor kidneys^26^,^27^. Therefore, these results demonstrated that HES accumulation in donor kidney is associated with the quality of the donor kidney as evaluated by the KDRI, and the graft renal function at 3 months. In addition the HES accumulation was positively and strongly correlated with the allograft renal function at 3 months (r = 0.88, p < 0.001); which is in accordance with this pathophysiological observation This finding illustrates the principle that the donor kidney quality can be assessed by means of the detection of an agent currently used in clinical practices, which is glomerular filtered, partially reabsorbed and stocked in the proximal tubule.

The main result of this study i.e. the association between high HES signal in renal tubular cells and better renal graft quality could appear conflicting with the renal toxicity of this molecule. Nonetheless, this work does not question the renal toxicity of HES; which is well reported in literature. Certainly, we reported that the more intense HES Raman signal, the better allograft function is; but this association is dependent on the renal graft quality. In other words, we did not explore the potential toxicity of HES on renal function but the association between HES tubular absorption and renal graft quality in donor resuscitation conditions. Indeed, we found that an increasing HES signal is also linked with a lower risk of graft failure; which is mainly related to limited donor morbidities and consequently better renal graft quality.

Clinical characteristics of renal graft recipient were certainly not responsible of the persistence and intensity of HES Raman signal because the molecule administration was prior to transplantation. Contrarily, characteristics of renal graft donor such as diabetes, hypertension, and vascular diseases are very important because they impact directly the renal graft function of the donor and consequently the performance of the tubular epithelial cells, particularly concerning their reabsorption function. KDRI score compiles the main of donor feature of renal quality graft and consequently reflecting the renal graft quality. This could explain the association between KDRI and HES Raman signal.

The severity of acute tubular necrosis (ATN) is related to several features among which lower perfusion during donor resuscitation and cold ischemia delay. Nonetheless, the role of ATN in HES clearance remains unclear.

Moreover, using univariate analysis we reported a significant association between the HES accumulation in kidney and the administrated dose of HES while, in this study, diabetes and hypertension were not associated with HES accumulation. Furthermore, by multivariate analysis, we reported that the significant correlation between HES Raman signal and eGFR was independent from other factors included HES administrated dose. However the fact that administration is more frequent in donor died of a traumatic cause than in donor died of a vascular reason could be a bias to interpret this analysis.

These data supported the link between the decrease of tubular reabsorptive work and ischemic conditions^28^. Consequently, for the same quantity of HES molecules, tubular cells in a good quality graft may be able to reabsorb more HES than for a bad quality graft.

Unfortunately, data about HES clearance were not accessible. However, it seems obvious that a better quality graft, with a better perfusion rate, should induce a higher HES clearance rate. Consequently, the fact we detected more HES Raman signal in better quality graft underlines the much greater amount of reabsorbed HES by tubular cells in these grafts than in poor quality grafts.

In this study led in the renal transplantation context, we investigated the relationships between microscopic tissue data probed by Raman imaging and clinical criteria used in medicine to assess the organ quality and consequently the organ function. Main published Raman studies demonstrated the potential of the technique to reveal, in various tissues, biomolecular alterations associated to a pathological state for diagnosis purposes^29^,^30^,^31^,^32^,^33^,^34^,^35^, or for investigating intracellular pharmacological characteristics of anticancer drugs^13^,^36^,^37^,^38^,^39^. In these works, the spectroscopic information were validated by confronting them to histopathological (conventional and immunochemical staining) or cell biological reference analysis. In our study, we considered a different and original approach by taking physiological indicators of the whole organ quality as reference criteria. Thus, Raman-based detection of an exogenous molecule appeared as a potential maker of the renal graft quality which is closely associated with renal graft function and survival. However much more work should be done before HES signal in kidney transplants could be used as clinical biomarker.

## Materials and Methods

### Study Patients

To obtain complete information about the colloids used, only the transplantations performed from brain-dead kidney donors managed within our institution were considered. In addition, to be included in the study, the selected kidney recipients must have had protocol graft biopsies at 3 months after transplantation (M3 protocol renal graft biopsies). Patients with acute allograft rejection in the first three months after transplantation were excluded. All experiments using human tissues samples were carried in accordance with relevant guideline and with the approval of the Institutional Review Board of CHU de Reims for ethics issues. An informed consent was obtained from all alive subjects. According to the HES administered to the donor during the procurement procedure, two groups were defined, namely: patients who received HES 130/0.4 (VOLUVEN; FRESENIUS-KABI, Sèvres, France) (HES group) and patients who did not receive HES solution (control group).

### Clinical and laboratory measurements

Recipient data included age, gender, and panel-reactive antibodies. Transplantation data included immunosuppressive treatment, HLA matching, history of borderline or acute allograft rejection, cold ischemia duration, delayed graft recovery (defined as absence of renal function, requiring dialysis, on one or more occasions within the first week after transplantation), estimated Glomerular Filtration Rate (eGFR) calculated by MDRD equation at M3^40^, M3 graft biopsy score according to the Banff 2009 classification^41^. The following donor characteristics were recorded to calculate the Kidney Donor Risk Index (KDRI): age, height, weight, ethnicity, history of hypertension, history of diabetes, cause of death, serum creatinine, HCV status, donation after cardiac death^27^. M3 protocol renal graft biopsies were re-analysed by an experienced nephropathologist to investigate the presence of tubular vacuolization and osmotic nephrosis-like lesions.

### Renal Graft biopsies

Frozen M3 protocol renal graft biopsies were used for spectral image acquisition. Sections 10 μm thick were cut from frozen biopsies by using a cryomicrotome. Samples to be analyzed were deposited on CaF_2_ substrates (CRYSTRAN, Dorset, UK) appropriate for near-infrared Raman spectroscopy. An independent set of 10 archived biopsy samples from native kidneys or from transplanted patients who never received HES were used as negative controls for HES spectral signal detection. These negative control biopsies comprised intravenous immunoglobulin-induced osmotic nephrosis-like lesions, two diabetic nephropathies, one chronic tubulo-interstitial nephropathy, one amyloid light-chain (AL) amyloidosis, one myeloma tubulopathy without amyloidosis, one oxalosis, one biopsy with tubular cell vacuolizations associated with calcineurin inhibitor toxicity, and two normal renal biopsies. These biopsy samples were selected from the tissue bank of Maison Blanche University Hospital (Reims, France).

### Raman acquisition parameters

Raman acquisitions were performed as previously described^16^ using a Labram Raman microspectrometer (HORIBA SCIENTIFIC, Villeneuve d’Ascq, France), equipped with a 785 nm near-infrared excitation source delivered by a Titanium-Sapphire laser. The analysis of the Raman signals was carried out using holographic dispersive grating (950 g/mm) and a CCD (Charge Coupled Device) camera making it possible to simultaneously measure several wavelengths in one shot. Thus, spectral data were collected on a spectral range from 400 to 1780 cm^−1^, with a spectral resolution of 4 cm^−1^. The spectrometer was coupled with an optical upright microscope (OLYMPUS, Bx40, Tokyo, Japan) equipped with a 100X objective (water immersion for HES solution analysis, NA = 1.35 or dry objective for tissue measurements, NA = 0.9). Acquisition parameters were controlled by Labspec software (HORIBA SCIENTIFIC, Villeneuve d’Ascq, France).

### Raman spectral acquisition of HES solution

For the HES 130/0.4, reference signature spectra of commercial solution and of the dehydrated form were collected with an acquisition time of 30 seconds and 3 accumulations per measurement. Photobleaching was performed for 5 to 10 seconds according to the fluorescence intensity of the sample. These conditions ensure recording high signal to noise ratio data. One hundred spectra were collected for each product to build a reference mean spectrum of HES 130/0.4 as previously described^20^.

### Raman spectral acquisition of kidney biopsies

In a preliminary study, we examined the intra-renal variability of HES Raman signal in tubular sections. In this view, we selected 3 distinct areas of tubular sections to perform Raman acquisitions and HES detection. Then we compared these areas to examine variability of HES Raman signal. We did not observe a significant difference about HES Raman signal in tubular sections between these different zones (data not shown). This result allowed us to lead the Raman analysis of our cohort on a single region of interest of tubular section.

For Raman acquisitions, 10-μm thick sections were cut from frozen biopsies by using a cryomicrotome. These sections were examined by an experimented renal pathologist under a white light microscope to localize the regions of interest. These regions were defined by the presence of tubular sections without glomerulus or vessels except peritubular capillaries. On such cryosections, the presence of microvacuolization of renal tubular epithelial cells cannot be affirmed due to the morphological degradation consecutive to the tissue congelation. Nonetheless, Masson trichrome staining of fixed fragments of these renal biopsies revealed the absence of vacuolization of tubular epithelial cells. The regions of interest of the tissue section were then mapped using a point-by-point image mode with a lateral displacement step of 1 μm in both the X and Y directions and an acquisition time of 45 s per pixel.

This method makes it possible to obtain a spectrum for each pixel analyzed. In our investigation for both negative controls and positive specimens, tubular section areas of about 500 μm^2^ were imaged.

### Data pre-processing and image analysis

The pre-processing of Raman spectral data was performed using LabSpec 5 software (HORIBA SCIENTIFIC, Villeneuve d’Ascq, France). The procedure was performed as follows: correction of instrument response in the spectra by subtracting the black current, detector response and optical setup signals, noise reduction using a 5 points average Savitsky–Golay smoothing and finally baseline correction using a polynomial function of degree 5 for removing the fluorescence background. This pre-processing was done on the whole spectral region (400–1800 cm^−1^) and spectra with low signal to noise ratio (SNR < 10) were discarded. The mean signal is calculated in –CH region (2800–3000/cm^−1^) and the noise in 2000–2200 cm^−1^.

The processing of corrected data maps was performed by using homemade software based named on least squares fitting method that operates in the Matlab environment (THE MATH WORKS INC., Natick, MA, USA). This method was named Fitting algorithm. A thorough description of this statistical analysis has been described elsewhere^16^,^42^. For spectral image processing, the set of spectra was vector normalized on the whole spectral range. Each pre-processed Raman spectrum can thus be described by the following linear model:


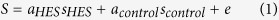


where *S* is the considered corrected Raman spectrum, *s*_*HES*_ and *s*_*control*_, respectively, corresponding to the reference Raman spectra of HES and untreated normal renal sample (negative control).

The coefficients *a*_*HES*_ and *a*_*control*_ correspond to the abundance fractions of each reference spectrum into the recorded spectrum *S. e* represents the modelization error.

The estimation of the fitting coefficients *a*_*HES*_ and *a*_*control*_ for each spectrum give access to the relative HES concentration in each pixel and leads to the construction of images reflecting the HES distribution into the renal graft sections.

For a spectral image, the result defined as HES spectral signal, was the given as the ratio of HES positive pixels to the total number of pixels. From an independent set of ten negative control biopsies, the mean and standard deviation (SD) values of the HES spectral signal were determined to be 0.12 ± 0.04. Consecutively, the threshold for HES spectral signal positivity was fixed as the mean value of these negative controls plus 2 SD, i.e. equal to 0.2. Raman microimaging analysis was blindly performed for M3 protocol renal graft biopsies originated from HES and control groups.

Quantification of the HES in tissue sample was recently demonstrated by our team [19]. Briefly, using the reference spectrum of HES 130/0.4, a fitting method was employed to quantify the relative contribution of the HES spectrum for each tissue pixel. For an image, the result was given as ratio of HES positive pixels to the total number of pixels. This ratio was considered as the HES spectral signal. The mean value of the HES spectral signal had been determined to be equal to 0.12 ± 0.04 for the independent set of ten negative control biopsies. The threshold for HES spectral signal positivity was therefore defined as the mean value of negative control +2 SD i.e. equal to 0.2. Raman spectroscopy microimaging acquisition and analysis were blindly performed for M3 protocol renal graft biopsies from the HES and control groups.

### Statistical analysis

Quantitative data were described as means ± standard deviation and qualitative variables as number and percentage. Univariate analysis was performed using the Student t test, Wilcoxon rank test, Pearson’s correlation test or Spearman’s correlation test, according the appropriated conditions. Multivariate analysis was performed to identify factors associated with the HES spectral signal, using stepwise linear regression, and including factors whose p-values were <0.10 by univariate analysis. Entry and exit thresholds were set at 0.20. A p value < 0.05 was considered as significant. All analyses were performed using IBM SPSS software version 20 (IBM, Armonk, NY, USA).

## Additional Information

**How to cite this article**: Vuiblet, V. *et al*. Raman-based detection of hydroxyethyl starch in kidney allograft biopsies as a potential marker of allograft quality in kidney transplant recipients. *Sci. Rep*. **6**, 33045; doi: 10.1038/srep33045 (2016).

## Figures and Tables

**Table 1:** Population characteristics.

Variables	HES (N = 20)	No HES (N = 6)	p
*Recipient characteristics*			
Age (mean ± SD)	45.75 ± 13.31	53.33 ± 13.45	0.23
Male sex (%)	40	50	0.66
*Donor characteristics*			
Age (mean ± SD)	45.70 ± 15.14	54.1 ± 23.26	0.30
Male sex (%)	60	66.7	0.78
Hypertension (%)	15	50	0.07
Diabetes (%)	5	0	0.57
Cause of death			
Vascular (%)	70	66.7	0.87
Trauma (%)	30	33.3	0.87
Cardiac arrest (%)	20	16.7	0.85
Serum creatinine (μM) (mean ± SD)	86.70 ± 35.85	79.17 ± 39	0.66
KDRI (mean ± SD)	1.01 ± 0.24	1.38 ± 0.64	0.22
*Graft characteristics*			
Cold ischemia duration (min) (mean ± SD)	974 ± 286	933 ± 288	0.79
Delayed graft recovery (%)	15	0	0.31
eGFR at M3 (ml/mn/1.73 m^2^) (mean ± SD)	56.88 ± 23.12	47.89 ± 16	0.38
IFTA at M3			
stage 0	85	100	0.31
stage 1	15	0	0.31
stage 2	0	0	0.31
stage 3	0	0	0.31
HES Raman signal (mean ± SD)	0.21 ± 0.27	0.05 ± 0.06	0.02

HES hydroxyethyl starch; KDRI, Kidney donor risk index; eGFR: estimated Glomerular Filtration Rate by MDRD equation, IFTA: Interstitial Fibrosis and Tubular Atrophy score from BANFF 2007 classification.

**Table 2:** Factors associated with HES Raman signal.

Variables	Univariate analysis			Multivariate analysis^b^
	HES signal^a^	Correlation coefficient (r)	p	p
*Recipient characteristics*				
Age		−0.53	0.01	0.44
Male sex (Y/N)	0.28/0.1		0.07	0.52
*Donor characteristics*				
Age		−0.72	<0.001	0.72
Male sex (Y/N)	0.18/0.16		0.85	
Hypertension (Y/N)	0.02/0.2		0.18	0.66
Diabetes (Y/N)	0.05/0.18		0.58	
Cause of death				
Vascular (Y/N)	0.09/0.4		0.03	0.61
Trauma (Y/N)	0.4/0.09		0.03	0.61
Cardiac arrest (Y/N)	0.2/0.17		0.81	
Serum creatinine		0.56	0.81	
Administered volume of HES		0.63	0.002	0.57
KDRI		−0.72	<0.001	0.54
*Graft characteristics*				
Cold ischemia duration		−0.24	0.3	
Delayed graft recovery	0.01/0.2		0.16	0.57
eGFR at M3		0.88	<0.001	0.006
IFTA at M3 (0/>0)	0.03/02		0.21	

^a^Values exposed are mean of HES signal in presence or absence of qualitative variable.

^b^For the multiple regression analysis, only the variables with one p < 0.20 were retained. Y: yes; N: No; HES hydroxyethyl starch; KDRI, Kidney donor risk index; eGFR: estimated Glomerular Filtration Rate by MDRD equation, IFTA: Interstitial Fibrosis and Tubular Atrophy score from BANFF 2007 classification.
